# Spatially Explicit Active Learning for Crop-Type Mapping from Satellite Image Time Series

**DOI:** 10.3390/s24072108

**Published:** 2024-03-26

**Authors:** Beatrice Kaijage, Mariana Belgiu, Wietske Bijker

**Affiliations:** Faculty of Geo-Information Science and Earth Observation, University of Twente, 7522 NH Enschede, The Netherlands; beatriceanth2006@gmail.com (B.K.); w.bijker@utwente.nl (W.B.)

**Keywords:** spatial autocorrelation, supervised classification, remote sensing, agriculture, scarce label environments

## Abstract

The availability of a sufficient number of annotated samples is one of the main challenges of the supervised methods used to classify crop types from remote sensing images. Creating these samples is time-consuming and costly. Active Learning (AL) offers a solution by streamlining sample annotation, resulting in more efficient training with less effort. Unfortunately, most of the developed AL methods overlook spatial information inherent in remote sensing images. We propose a novel spatially explicit AL that uses the semi-variogram to identify and discard redundant, spatially adjacent samples. It was evaluated using Random Forest (RF) and Sentinel-2 Satellite Image Time Series in two study areas from the Netherlands and Belgium. In the Netherlands, the spatially explicit AL selected 97 samples achieving an overall accuracy of 80%, compared to traditional AL selecting 169 samples with 82% overall accuracy. In Belgium, spatially explicit AL selected 223 samples and obtained 60% overall accuracy, while traditional AL selected 327 samples and obtained an overall accuracy of 63%. We concluded that the developed AL method helped RF achieve a good performance mostly for the classes consisting of individual crops with a relatively distinctive growth pattern such as sugar beets or cereals. Aggregated classes such as ‘fruits and nuts’ posed, however, a challenge.

## 1. Introduction

Supervised machine learning has experienced tremendous progress in recent years being increasingly used for classifying crop types from Satellite Image Time Series (SITS) [1,2]. Although deep learning classifiers can successfully identify crops from SITS without requiring the involvement of the image analysts in the selection of the input features [3,4,5,6,7,8], traditional supervised machine learning remains a viable alternative [9,10,11]. Machine learning methods learn the characteristics of the target crops from training samples collected through intense field campaigns or expert-based interpretation of very high-resolution satellite images [12]. Unfortunately, annotating a large number of samples is a time-consuming and expensive task [13]. Consequently, different solutions have been proposed to address the challenges associated with the availability of samples: (1) generating labeled crop samples for the target classification years by using samples from previous years [10]; (2) developing (semi-)automatic solutions to label crop samples [13]; (3) using classifiers such as Dynamic Time Warping that require a small number of training samples [14,15]; (4) leveraging the information learned by supervised methods in areas where many labeled samples are available through transfer learning methods [16,17]; and (5) reducing the number of crop samples to be annotated without decreasing the performance of the supervised methods [18]. This means that the training samples remain representative of the statistical distribution of data [19].

Active Learning (AL) is one of the solutions to reduce the number of samples to be annotated by several orders of magnitude. It helps to select the most informative samples to be labeled from a large unlabeled sample set [20]. Two criteria are commonly used to rank the candidate samples relative to their potential contribution to the classification problem: uncertainty and diversity. The uncertainty criterion aims to identify the samples that pose challenges to the supervised methods. The uncertainty of a sample can be quantified using margin sampling [21], disagreement between a committee of classifiers [22], posterior probabilities of class membership [23], or entropy [24]. Since the samples selected based on uncertainty alone may not be well representative of the classes present in the dataset, the diversity criterion has been introduced in AL studies. Diversity measures ensure class representativeness among selected samples. These measures include density-weighted metrics or clustering-based approaches [18]. Euclidean distance or cosine similarity metrics are the most common similarity measures used to select the most dissimilar samples [25].

AL has been successfully implemented in several remote sensing applications including land cover classification with Support Vector Machine and multispectral images [26], improvement of land cover classification from hyperspectral images using both traditional AL [23] or AL that accounts for spatial information [27], urban land cover-land use classification through object-based image analysis [28]. In terms of application domains, AL has been used for biophysical parameter estimation [29], tree species mapping [30], crop area mapping [31], crop type mapping using satellite image time series [32] and hyperspectral images [33], large-extent cultivated area mapping [31], and poplar plantation mapping at the national level [18]. Recently, AL has been implemented to reduce the annotation efforts required by various deep learning algorithms [34,35].

Previous studies focused mainly on AL heuristics that use spectral data to optimize the collection of samples to be annotated while ignoring the spatial information inherent in remote sensing images. Nonetheless, AL-based selected samples may be spatially contiguous and this might result in a redundant sample set. A limited number of studies accounted for the spatial distribution of samples when implementing AL algorithms. For example, Demir, et al. [36] included topography and road networks to reduce travel time when limited resources are available to annotate training samples. Zhang, Pasolli, and Crawford [33] showed the advantages of both spectral and spatial features extracted from segmentation maps in the proposed multi-view AL method to map crops from hyperspectral images. Pasolli, et al. [37] used the Euclidean distance, Parzen window method, and spatial entropy as spatial criteria to ensure that the selected samples are spatially representative of the entire study area. The authors combined spectral and spatial criteria using nondominated sorting. Calculating spatial autocorrelation between samples within an area is an alternative approach for identifying redundant samples in the space domain. Different measures can be used to quantify the spatial autocorrelation including Moran’s I [38], Geary’s C ratio [39], or the semi-variogram. Moran’s I and Geary’s C are global spatial autocorrelation measures, whereas the semi-variogram shows how spatial variation changes as a function of the distance between point location pairs. Stumpf, et al. [40] developed a region-based AL method for landslide mapping that reduces the samples to be annotated to those situated in a few compact spatial batches. The authors measured the spatial autocorrelation of gray values within input images using a semi-variogram analysis to identify the minimum size of sampling windows to capture “spatial variability beyond locally autocorrelated characteristics”.

In our study, we propose the semi-variogram to compute the spatial autocorrelation between training samples and discard neighboring samples from the unlabeled but representative sample set selected using an AL method applied for crop type mapping from SITS. The method, referred to as spatially explicit AL, eliminates redundant samples while achieving results comparable to those obtained by the supervised classifier trained with a larger number of randomly selected samples. Our method was implemented using a Random Forest (RF) classifier and tested in two study areas from the Netherlands and Belgium. We compared our results to the traditional AL approach that does not exploit spatial information to select the most representative samples for training.

The remainder of the paper is organized as follows: Section 2 introduces the two study areas, and the Sentinel-2 SITS used to identify the target crop types. Section 3 is dedicated to the adopted methodology where we are introducing the concept of the spatially explicit AL method proposed in our study. Section 4 and Section 5 focus on the presentation of the results and their interpretation. The paper ends with conclusions in Section 6.

## 2. Study Areas and Datasets

**Figure 1:**
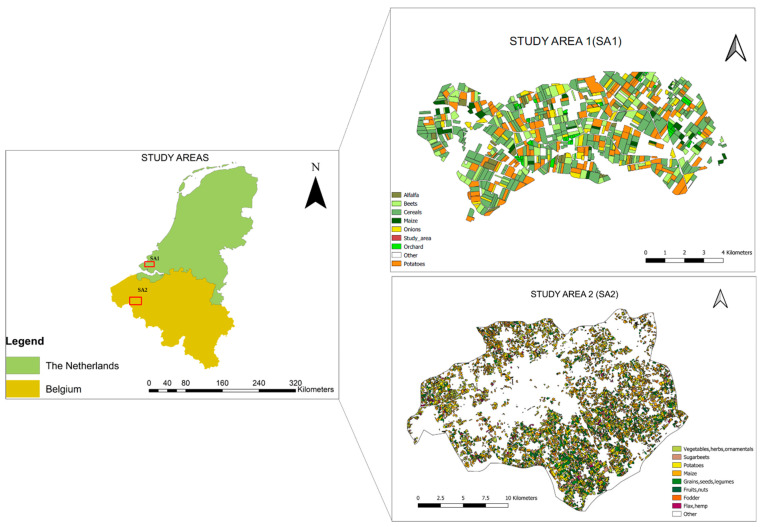
Spatial location of the two investigated study areas: Study Area 1 (SA1) and Study Area 2 (SA2)—including the crop type distribution in these areas.

Our research was conducted in two study areas with agricultural land use (Figure 1). Study Area 1 (SA1) is located in Noord Beveland, a municipality in the province of Zeeland in the Southwest of The Netherlands. Being a polder resulting from land reclamation, the area yields fertile soils, and large and regularly shaped parcels, making it suitable for agriculture. Study Area 2 (SA2) is situated in the municipality of Kortrijk (Flemish), also known as Courtrai (French/English).

The area is located in the Flanders Region in the western part of Belgium, along the River Leie (Lys) and the Leie–Scheldt Canal. It is one of the important agricultural areas in the country and contains many farming households. The agriculture parcels in Kortrijk are much smaller than those from Noord Beveland. To implement the proposed method, we used the existing datasets on the cultivated crops in the two selected study areas. A total of 54 crop classes for SA1 and 13 crop classes for SA2 were available. However, we reduced the classes to seven crops for SA1 and eight crop classes for SA2, omitting the classes with less than five parcels. For SA1, the crop data were obtained from the Base Registration Crop Parcels agency in the Netherlands (www.PDOK.nl (accessed on 21 March 2024)), and the parcel boundaries from the Agricultural Area of the Netherlands (AAN). The 1584 parcels available in the investigated study area represent arable land, grassland, natural area, and fallow land. Our research focused solely on the 951 crop parcels and seven crop types: cereals; potatoes; beets; onions; orchard; maize; and alfalfa (Table 1). The remaining land use/land cover classes were grouped into ‘water’ and ‘other’. For SA2, the crop data were acquired from the Flemish geoportal Geopunt (www.geopunt.be (accessed on 21 March 2024)). The data consist of agricultural parcels associated with cultivated crops. Eight crop classes were used for the research: maize; grains, seeds, and legumes; potatoes; vegetables, herbs, and ornamental plants; sugar beets; fodder; flax and hemp; and fruits and nuts (Table 2). An additional class of ‘other’ areas was added incorporating the remaining land use and land cover classes available in the study area.

For SA1, we used the crop data from 2019, whereas for SA2 we used the data from 2018. The selection of the year was contingent on the cloud coverage. According to the Royal Meteorological Institute (KNMI) in the Netherlands, 2019 was a very warm, sunny, and quite dry year on average, and a decrease in the rainfall compared to the long-term monthly average was observed in the southwestern part of the country where SA1 is located. On 25 July, the Netherlands had its highest temperature in at least three centuries. This was a time when most of the crops were at their peak growth. Both May and November were unusually cool. These were sowing and harvesting months for maize, potatoes, and onions. In the case of SA2, 2018 was a dry year, with a maximum of 37 °C on the 27 of July and of 36 °C on the 7th of August. The meteorological information for SA1 was obtained from the nearby weather station of Vlissingen via the national meteorological service of KNMI, and the information for Kortrijk from the weather station of Kortrijk via (weer1.com (accessed on 21 March 2024)).

## 3. Methods

**Figure 2:**
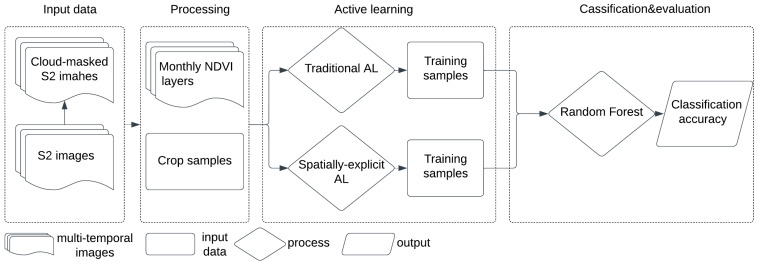
Workflow of the methodology proposed in this study. S2: Sentinel-2 images; NDVI: Normalized Difference Vegetation Index; AL: Active Learning; Traditional AL: AL using solely spectral information; Spatially explicit AL: AL using spatial and spectral information.

The main methodological steps include (i) image processing; (ii) sample generation for SA1 and SA2. (Note that for our study, we used the existing crop-type database to label the samples selected through AL. In the regions where these data are missing, the users need to label the data through field campaigns.); (iii) AL-based training sample selection considering the spectral domain only; (iv) development of a spatially explicit AL strategy using a semi-variogram; (v) crop type classification, first using a larger number of training samples, then using only samples identified as being relevant by spectral-based AL and, finally, using only training samples generated by spatially explicit AL, and (vi) evaluation of the obtained results (Figure 2).

### 3.1. Satellite Image Pre-Processing

**Figure 3:**
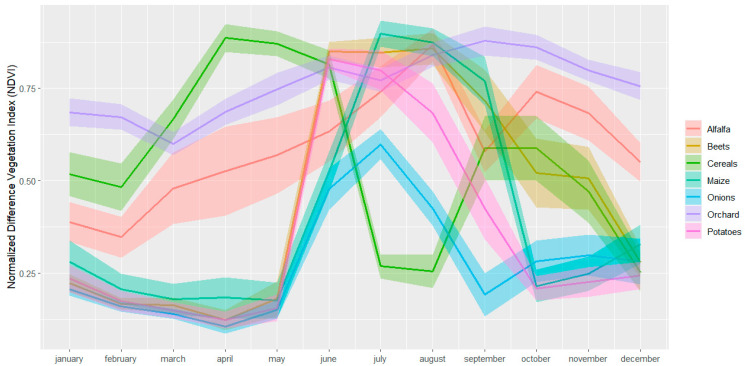
Temporal profiles and standard deviation of the target crops in SA1 calculated using Normalized Difference Vegetation Index (NDVI) Satellite Image Time Series (SITS).

**Figure 4:**
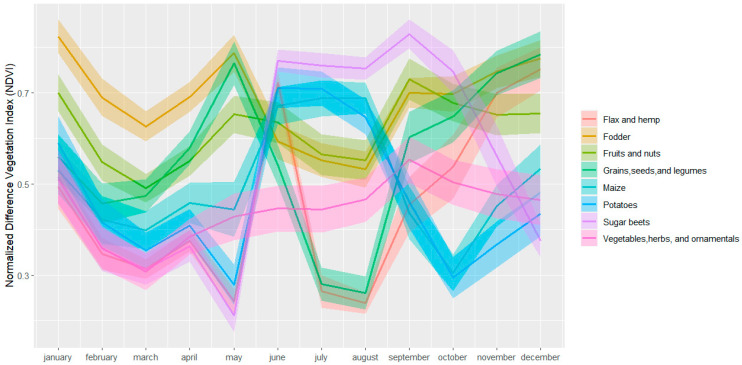
Temporal profiles and standard deviation of the target crops in SA2 calculated using Normalized Difference Vegetation Index (NDVI) Satellite Image Time Series (SITS).

The SITS used as input in our research consists of a monthly time series of Sentinel-2 images. The clouds were masked out using the QA60 band. In the next step, the Normalized Difference Vegetation Index (NDVI) for each Sentinel-2 image was computed using bands 8 and 4. The temporal profiles of the target crops representing the average of 50 randomly selected samples in SA1 and 70 randomly selected samples in SA2 are presented in Figure 3 and Figure 4.

### 3.2. Training Sample Preparation

Using the crop parcels described in Table 1 and Table 2, 630 point-based samples were generated for the investigated target classes in SA1 and 900 point-based samples for the target classes in SA2. For SA1, we selected 70 samples per class using a stratified random sampling approach, whereas for SA2 we applied the same sampling strategies but selected 100 samples per class. The number of samples for the two investigated study areas differs because SA2 has more aggregated classes than SA1 (Figure 3) which increases the inter-class variations as depicted in Figure 4 for ‘vegetables, herbs, and ornamentals’, ‘fruits and nuts’ and ‘flax and hemp’. Consequently, a larger number of samples is required to represent these high variations. The samples were further divided into training (70%) and testing samples (30%). The training and testing sample sets were sampled from different crop parcels to ensure their spatial independence and, in this way, to reduce the risk of overestimating the classification performance [3,41].

### 3.3. Selection of Training Samples Using Active Learning

The components of an active learner consist of a set of classifiers *C* trained on a small set of labeled samples *L*, a query *Q*, implemented to identify and select the most informative, i.e., uncertain, labels from a set of unlabeled samples *U* which are not annotated yet and a supervisor *S*, assigning labels to the retrieved samples [42]. The procedure is initialized by training the selected supervised classifier using the sample set L. In the next step, the query *Q* identifies and selects the most informative samples from *U* using a user-defined criterion or several criteria. Lastly, supervisor S annotates the retrieved unlabeled samples that are further added to the L set and used by the supervised classifier for retraining. This is an iterative procedure that continues until it reaches a user-defined stopping criterion that can, for example, be defined based on the classification accuracy [43].

Two AL scenarios were implemented in this research. The first AL scenario uses spectral domain heuristics to query informative samples and is referred to in this paper as spectral-based AL. These heuristics query unlabeled samples using only their characteristics in the feature space. As mentioned in the introduction section, some of these heuristics are posterior probability, least confidence, margin sampling, and entropy [18]. In our work, we propose using Query By Committee (QBC), where a user-defined number of learners are trained from a pool of labeled samples, and each learner, i.e., committee member, votes for the label to which a potential sample belongs. The sample for which most of the implemented learners of the committee disagree is selected. This method is less computationally intensive than other AL approaches [40]. Since a small number of committee members proved to be efficient in the previous work dedicated to AL [44,45], we used two committee members represented by two RF classifiers [46].

We divided the 350 samples available for SA1 into a labeled sample set *L* consisting of 40 samples and an unlabeled set *U* containing 310 samples. For SA2, 40 samples (*L*) out of 560 available samples were used for initializing the training. In both cases, the *L* samples were randomly selected. In the next step, the informative unlabeled samples were queried from *U* and the performance of the committee was assessed with an increment of the number of training samples used to train the model. The prediction accuracy of the committee was assessed by comparing the predicted class label with the known class label of the sample. NDVI values of each unlabeled sample were used to determine its importance using vote entropy (Equation (1)) as a metric for the amount of disagreement between committee members. As expressed by

(1) $$x_{VE}^{*}=\underset{x}{\mathrm{argmax}}-\sum_{i}\frac{V_{y_{i}}}{C}\log\frac{V_{y_{i}}}{C}$$

where $x_{VE}^{*}=\mathrm{vote}\mathrm{entropy}\mathrm{for}\mathrm{sample}x;$ *y_i_* = possible labels; *Vy_i_* = number of votes received from the committee members; and *C* = number of members/committee size.

In the second AL scenario, the selection of the informative samples accounted for their characteristics both in the feature space and in spatial information. This implementation is referred to as spatially explicit AL throughout our paper and it accounts for the spatial distribution of the samples assessed by spatial autocorrelation measures. Spatial autocorrelation describes the spatial dependency between objects (or variables) and is an expression of how similarity between objects (or variables) depends on their relative position [47]. It can be quantified by global or local measures or by a semi-variogram. Global measures, such as Moran’s I [38] or Geary’s C ratio [39] summarize the level of clustering across the entire area of interest in one single statistic without identifying *where* in the area the similarity occurs. For our work, it is crucial to understand *where* the (dis)similarity occurs to select the most relevant, i.e., most informative samples. Therefore, global measures cannot help. Local measures, such as the Local Indicator of Spatial Association (LISA) [48] and local Moran’s I [49], indicate the location of clusters explained by the overall global pattern and give a single statistic for each locality. However, the current research needs a measure of spatial autocorrelation at the sample level, on a point-to-point basis. The semi-variogram is a function relating semi-variance to sampling lag, i.e., the distance between samples [50], and can be used as a characterization of the spatial structure of an area. When the semi-variance is plotted against the distance between samples, the semi-variance typically increases until it reaches a plateau. The distance at which the plateau is reached is called the range and the semi-variance at that point is called the sill, i.e., the total variability in the data. The semi-variance when the distance between points is zero is called the nugget and quantifies the randomness of the data [51], accounting for measurement errors and non-spatial variability.

Only sample pairs further apart than the range can be considered spatially uncorrelated. Therefore, only samples further apart than the range were considered in the spatial component for AL. Different semi-variogram models, namely spherical, exponential, and Gaussian models were used to model the semi-variogram and capture the spatial variability of data with distance. The model with the smallest Sum of Squares Error (SSErr), considering both test areas, was chosen as the best-fitting model. Semi-variograms based on NDVI values were estimated for each month for both study areas, using at least 30 pairs of points for each lag distance to obtain a reliable estimate. After several runs, the spherical model gave the smallest SSErr for most of the semi-variograms. For an impression of the varying ranges and nuggets of the fitted models, the values for each month are given in Table 3 for SA1. The range and nugget of the monthly models were used to select the semi-variogram model for the new spatial component in AL. For each month, the semi-variogram model with the lowest nugget value was chosen. For each study area, from the resulting 12 semi-variograms of the 12 months, the smallest range distance was chosen as the minimum distance for selecting spatially informative samples, i.e., the minimum distance above which samples can be considered uncorrelated. Choosing larger range distances would risk losing potentially informative samples, also this is unfeasible because of area size limitations. For this reason, the chosen ranges were 417.6 m for SA1 and 529 m for SA2.

Therefore, all samples with an in-between distance above these ranges were considered spatially informative. The learner first queries an informative sample using the entropy and then assesses the Euclidean distance between the selected sample and the label set. If any of the Euclidean distances between the selected samples and the labeled sample are below the semi-variogram range, the point is discarded. The spatially explicit AL method was implemented to select the most informative samples according to vote entropy and spatial autocorrelation. For both spectral-based and spatially explicit AL, we used a stopping criterion that accounts for the increase in the prediction accuracy with each added sample. Therefore, when the accuracy leveled off, the sample selection procedure stopped.

### 3.4. Crop Type Classification Using the Random Forest Classifier

Image classification was performed using RF [46]. RF is a very popular classifier in remote sensing due to its high performance [52]. It consists of several internal decision trees built using a randomly selected subset of features and training samples selected randomly through replacement (Breiman, 2001 [46]). This way of selecting training samples and input features minimizes the correlation between the built decision trees [53]. The classifier used about 2/3 of the labeled samples for training, and the remaining 1/3 of the samples, called Out of Bag (OOB) samples, were used to assess the classification accuracy (Breiman, 2001 [46]). RF is mainly sensitive to the number of trees (*ntree*) and the number of selected input features (*mtry*). The *ntree* was set to 1000, whereas *mtry* was defined as being the square root of the total number of input features. Fifty iterations were defined for each RF model. The reported accuracies were averaged across these iterations. An RF-based classification was implemented using the NDVI image stack as an input feature for both study areas. It was first trained using all available annotated samples and then by using training samples generated from the two AL implementations: spectral-based AL and spatially explicit AL. The classification results were assessed using the Kappa coefficient, overall accuracy (OA), User’s Accuracy (UA), and Producer’s Accuracy (PA) [54,55,56].

## 4. Results

### 4.1. Active Learning Results

In the case of spectral-based AL, the accuracy stopped increasing further after 129 queries, when committee prediction accuracy reached 99.43% for SA1. A total of 169 crop-type samples (40 initial samples plus 129 newly selected samples) were selected through this AL strategy (Table 4). This represents 48% of the entire sample set (350 crop-type samples), excluding water and other classes. For SA2, the accuracy leveled off after 287 queries, with a committee prediction accuracy of 95.2%. In this case, a total of 327 samples were selected (40 initial samples plus 287 newly selected samples) (Table 5). This represents 58% of all available samples excluding the ‘other’ class.

When using spatially explicit AL, the accuracy leveled off after 57 queries with a committee prediction accuracy of 90.9% in SA1. Thus, the labeled sample pool consists of a total of 97 samples (57 newly selected samples plus the 40 initial samples). This represents 28% of the entire available training sample set. For SA2, the accuracy stopped increasing further after 183 queries with a committee prediction accuracy of 82.14%. A total number of 223 informative samples were selected representing 40% of the entire training sample set. Table 1 and Table 2 show the sample distribution across target crop types after selecting informative samples using both AL methods. The samples were not well balanced across target crop classes, and classes with high interclass similarity, e.g., potatoes and maize classes from both areas, were given preference in the selection. The distribution of samples per class for both study areas is presented in Table 4 and Table 5.

### 4.2. Image Classification Results Obtained Using Various Training Sample Sets

Four image classification scenarios were tested: Scenario 1—classification using the total number of the available training samples; Scenario 2—classification using training samples selected by spectral-based AL; Scenario 3—classification using training samples selected by spatially explicit AL; and Scenario 4—classification using a random selection of training samples from the labeled sample set, equal to the number of samples selected by spatially explicit AL. This last experiment was designed to evaluate the benefit of using the developed AL method to select the most informative training samples over selecting the same number of samples randomly.

The accuracies obtained for scenario 1 are presented in Table 6 and Table 7. For SA1, the entire 350 crop samples were used for classification obtaining an overall accuracy of 84% (Table 6). For SA2, a total of 560 training crop samples were used and yielded an overall accuracy of 65% (Table 7). The total number of testing samples used for this task was 270.

In the case of the second scenario for SA1, the overall accuracy was 82%. For SA2, there were a total of 327 samples, which gave an overall accuracy of 63%. For the third scenario in SA1, the overall accuracy was 80%. For SA2, 223-AL generated samples were used, which gave an overall accuracy of 60%. The fourth and last classification scenario was dedicated to randomly selecting the same number of samples as obtained by using AL and spectral and spatial components. An overall accuracy of 70% was attained for SA1. For SA2, an overall accuracy of 54% was obtained.

The UA and PA accuracies for all crop-type classes are displayed in Table 8 and Table 9 for SA1 and SA2, respectively.

The accuracies were high for SA1. In SA2, on the other hand, there was much more variation in the accuracies: some classes had high PA in all three scenarios (grains, seeds, legumes; and sugar beets), while others had moderate accuracies (flax and hemp; maize; potatoes; and vegetables, herbs, and ornamentals) or even a low producer accuracy (fodder; and fruits and nuts).

The proposed AL method yielded promising results for cereals (UA 90% and PA 95%), alfalfa (UA 86% and PA 95%), and onions (UA 84% and PA 80%), for SA1. The developed method also performed well in aggregated classes like ‘grain, seeds, and legumes’ (UA of 87% and PA of 87%) and the ‘fruits and nuts’ classes (UA of 60% and PA of 30%) as compared to the other approaches. For the ‘vegetables, herbs, and ornamentals’ class, the increase in UA was outweighed by a larger decrease in the PA.

## 5. Discussion

The overall goal of our work was to assess the potential of a spatially explicit AL strategy for selecting the most informative samples for crop-type mapping from SITS. The goal of this method is to select the smallest number of samples required to achieve good classification results. We showed that our spatially explicit AL strategy provides a high potential for decreasing the time and effort required for sample annotation in the two study areas. Pasolli et al. (2011) [19] have also emphasized that integrating a spatially explicit AL in a single-date image classification task obtained higher accuracy than the AL strategy that solely accounts for spectral information. While Pasolli et al. (2011) [19] used the Euclidean distance between the unlabeled training samples and support vectors (SVs) to select samples that are covered by SVs, we proposed a semi-variogram to discard redundant samples. Stumpf, Lachiche, Malet, Kerle, and Puissant [40] also used the semi-variogram, but with a different aim: to avoid spatially dispersed distribution of landslide samples that would otherwise increase the time and costs for field surveys and/or visual interpretation required to annotate the labels.

Reducing the size of the labeled set makes it more difficult to distinguish the subtle differences between spectrally similar classes. Several classes in SA1 and SA2 showed high inter-class similarities (Figure 3 and Figure 4). For example, in SA1, the NDVI time-series-based crop growth patterns are similar between maize and potatoes. In contrast to these classes, alfalfa, cereals, and orchard show distinct temporal behaviors. In SA2, maize and potatoes also have similar crop growth patterns (see Figure 4) which makes them challenging to distinguish. Previous studies have also highlighted the difficulties of successfully distinguishing crop types with high inter-class similarities like maize and potatoes [13,14]. Sugar beets or ‘grain, seeds, and legumes’, on the other hand, have a more distinct growth pattern and consequently, there is less overlap with the other crops and less confusion and misclassification.

In general, in SA2, accuracies were lower than in SA1 for all classes and in all scenarios. The lower classification accuracy and the smaller reduction in the number of training samples required for SA2 could be because most of the training sample classes are aggregated classes, with high intra-class variation hence causing a challenge in the classification process.

The spatially explicit AL method proposed in our study requires the selection of the range identified by the semi-variogram. The pairs of samples are spatially uncorrelated if the in-between distance is larger than the defined range. The selected samples depend highly on the capability of the implemented semi-variogram to identify the proper range which in turn helps us to select the most informative samples. If, for example, the range is too low, redundant samples might still be present in the sample pool. If the range is too large, the method might discard samples that are informative and essential to achieving good performance. Spatial Simulated Annealing (SSA) [57,58] could be used to optimize the spatial sample design.

Spatial heterogeneity caused by variations in environmental conditions and agricultural management practices might lead to variations in crop diversity and crop representation in the feature space. Therefore, applying the developed AL method to agricultural regions with multiple spatial patterns, e.g., containing a flat area with large parcels and a sloping area with smaller, irregular parcels might be challenging since the samples used to train the machine learning algorithms have to capture the environmental and management conditions that operate at different geographic scales. In areas with multiple spatial patterns, multiple semi-variograms, e.g., per sub-area, need to be generated to capture the spatial variability. In this way, it becomes difficult to select the most suitable range from several ranges generated by the sub-area semi-variograms.

In our work, we used the already annotated samples to test the efficiency of the proposed spatially explicit AL method. Yet, in many areas across the globe, the annotated labels are missing. In this situation, the samples need to be labeled either through visual interpretation, given that very high-resolution images are available, or through intense field campaigns. Our method brings benefits to these scenarios as well since the number of samples and, hence, the time required to annotate them is considerably reduced.

The proposed AL method will benefit future remote sensing-based applications in situations when the researchers can sample only a few locations for time and budget-related restrictions. Contrary to previous studies emphasizing the negative impact of insufficient training samples on the classification results [40], our study revealed the importance of spectral and spatial informativeness of samples in implementing classifiers with high performance.

In addition, this method works well when the training sampling is well-designed. In the case of SA2, almost all reference classes were aggregated classes, which led to low classification accuracy even when the entire dataset was used. This low accuracy was carried on to the developed spatially explicit AL strategy. Despite the overall low accuracy achieved in SA2, the conclusion that less than half of the training samples can be used to achieve similar accuracy if sample selection is based on selecting the spectrally and spatially most informative samples holds for both areas.

Since collecting labeled training samples is an expensive, time-consuming, and laborious task, there are only a few countries where crop-type benchmark datasets are available [59]. Future studies should focus on testing the proposed method in areas where annotated crop samples are limited such as developing countries.

## 6. Conclusions

This study demonstrated the importance of training sample selection, showing that by selecting spectrally and spatially informative samples, the number of training samples could be reduced to less than half while obtaining similar accuracies. This result was obtained in an area with large, regular parcels and classes consisting of single crops (SA1) as well as for an area with smaller parcels and aggregated classes consisting of several different crops (SA2). For SA1, the overall classification accuracies were 84% using all available 350 training samples, 82% using 169 samples selected using spectral-based AL, and 80% using 97 samples selected using spatially explicit AL. Selecting 97 samples randomly, without using spectral and/or spatial characteristics, yielded an average accuracy of 70%; therefore, the spatially explicit method gave a higher accuracy for the same number of samples. For SA2, the overall classification accuracies were 65% using all 560 training samples, 63% using 327 samples selected through traditional AL, and 60% using 223 samples selected using spatially explicit AL. Selecting 223 samples randomly resulted in an average accuracy of 54%.

The proposed AL method reveals that accounting for spatial information is an efficient solution to map target crops since it facilitates high accuracy with a low number of samples and, consequently, lower computational resources and time and financial resources for annotation. Future studies could extend the proposed method to different land cover mapping tasks. In addition, further research on the use of semi-variograms could be carried out for sampling design in areas containing sub-areas with different spatial variations caused by varying environmental conditions and management practices. Instead of the semi-variogram, other methods could be used for the spatial sample design, such as SSA.

## Figures and Tables

**Table 1:** Total number of crop parcels available in SA1.

Crop Type	Number of Crop Parcels
Cereals	305
Potatoes	157
Beets	117
Onion	68
Orchard	60
Maize	35
Alfalfa	27

**Table 2:** Total number of crop parcels in SA2.

Crop Type	Number of Crop Parcels
Maize	2989
Grains, seeds, and legumes	1533
Potatoes	1386
Vegetables, herbs, and ornamental plants	1365
Sugar beets	573
Fodder	273
Flax and hemp	96
Fruits and nuts	93

**Table 3:** Variation of the ranges and nugget values for the crop type samples cultivated in SA1 calculated using semi-variograms fitted for each month. The range identified for May has been selected to define the minimum distance for selecting spatially informative samples.

Month	Range (m)	Nugget
January	462.3	0.008
February	477.4	0.005
March	611.2	0.029
April	513	0.029
**May**	**417.6**	**0.025**
June	587.3	0.008
July	905.4	0.043
August	721.1	0.050
September	804.9	0.043
October	733.6	0.033
November	735.3	0.028
December	794	0.018

**Table 4:** Distribution of training samples per class before applying AL and after applying spectral-based AL and spatially explicit AL in SA1.

Crop Type	Total # of Training Samples	# of Training Samples Selected Using Spectral-Based AL	# of Training Samples Using Spatially Explicit AL
Alfalfa	50	22	11
Beets	50	28	16
Cereals	50	15	8
Maize	50	25	15
Onions	50	23	16
Orchard	50	15	7
Potatoes	50	41	24
Total	350	169	97

**Table 5:** Distribution of training samples per class before applying AL and after applying spectral-based AL and spatially explicit AL in SA2.

Crop Type	Total # of Training Samples	# of Training Samples Selected Using Spectral-Based AL	# of Training Samples Using Spatially Explicit AL
Flax and hemp	70	25	20
Fruits and nuts	70	40	25
Fodder	70	36	25
Grains, seeds, and legumes	70	32	21
Maize	70	56	41
Potatoes	70	58	39
Beets	70	33	30
Vegetables, herbs, and ornamentals	70	47	22
Total	560	327	223

**Table 6:** Classification results obtained using different classification scenarios developed for SA1: 1—classification results obtained using all samples; 2—classification results obtained using spectral-based AL; 3—classification results obtained using spatially explicit AL; and 4—classification results obtained using 97 randomly selected samples.

Classification Scenarios	# Samples	Kappa Index	Overall Accuracy (%)
Scenario 1	350	0.82	84
Scenario 2	169	0.79	82
Scenario 3	97	0.78	80
Scenario 4	97	0.66	70

**Table 7:** Classification results obtained using different classification scenarios developed for SA2: 1—classification results obtained using all samples; 2—classification results obtained using spectral-based AL; 3—classification results obtained using spatially explicit AL; and 4—classification results obtained using 223 randomly selected samples.

Classification Scenarios	# Number	Kappa Index	Overall Accuracy (%)
Scenario 1	560	0.60	65
Scenario 2	327	0.58	63
Scenario 3	223	0.55	60
Scenario 4	223	0.48	54

**Table 8:** Comparison of the User’s (UA) and Producer’s (PA) accuracies of crop types calculated and depicted in % for SA1.

Crop Type	Scenario 1		Scenario 2		Scenario 3	
	UA	PA	UA	PA	UA	PA
Alfalfa	100	95	100	85	86	95
Beets	75	75	65	75	71	75
Cereals	89	85	79	95	90	95
Maize	93	70	93	70	94	75
Onions	94	75	100	75	84	80
Orchard	75	90	87	95	66	95
Potatoes	77	85	68	75	71	75
Water	100	100	100	100	100	100
Other	64	80	76	65	56	30

**Table 9:** Comparison of UA and PA of crop types in % accuracies for the three scenarios for SA2.

Crop Type	Scenario 1		Scenario 2		Scenario 3	
	UA	PA	UA	PA	UA	PA
Flax and hemp	71	83	74	83	67	87
Fodder	39	30	41	30	38	33
Fruits and nuts	59	33	44	27	60	30
Grains, seeds, legumes	87	87	77	90	87	87
Maize	69	60	67	60	49	63
Potatoes	50	53	51	60	46	60
Sugar beets	63	80	66	77	63	73
Vegetables, herbs, ornamentals	63	67	59	57	75	10
Other	73	90	71	83	65	93

## Data Availability

Publicly available datasets have been used for this research.
